# Optimized design of surgical steps in pars plana vitrectomy for macular hole retinal detachment in pathological myopia decreases rate of iatrogenic retinal break and shortens length of operation

**DOI:** 10.1186/s12886-023-02907-3

**Published:** 2023-04-11

**Authors:** Ying Zhu, Huizhuo Xu, Xianggui Wang

**Affiliations:** 1 Eye Center of Xiangya Hospital, Central South University, 87 Xiangya Road, Changsha, Hunan China; 2 Hunan Key Laboratory of Ophthalmology, Changsha, Hunan China; 3 National Clinical Research Center for Geriatric Disorders, Xiangya Hospital, Central South University, Changsha, Hunan China

**Keywords:** Surgical steps optimization, Iatrogenic retinal tear, Length of operation, Internal limiting membrane flap, Macular hole retinal detachment

## Abstract

**Background:**

To investigate the effect of surgical steps optimization in pars plana vitrectomy (PPV) with internal limiting membrane (ILM) flap for macular hole retinal detachment (MHRD) in pathological myopia.

**Methods:**

A retrospective, consecutive, nonrandomized comparative study. High myopic eyes diagnosed with MHRD receiving PPV with ILM flap from March 2019 to June 2020 in Department of Ophthalmology, Xiangya Hospital, Central South University were included in the study. Patients were included into two groups based on different design of surgical steps. In the routine group, extension of posterior vitreous detachment (PVD) towards periphery was performed right after induction of PVD. In the experiment group, the retina was reattached with drainage of subretinal fluid through macular hole before peripheral vitreous was dealt with. Complete ophthalmic examinations were performed before and after surgery. The follow-up time was at least 6 months. The rate of iatrogenic retinal break and length of operation were compared between the two groups.

**Results:**

Thirty-one eyes from 31 patients were included in the study with 15 in the experiment group and 16 in the routine group. Demographics showed no statistically significant difference between the two groups. Post-op BCVA, rate of macular hole closure and rate of retinal reattachment were similar in the two groups. The rate of iatrogenic retinal break in the experiment group was significantly lower than that in the routine group (6.7% vs. 37.5%, *P* < 0.05). The average length of operation was 78.6 ± 18.8 min in the routine group and 64.0 ± 12.1 min in the experiment group (*P* < 0.05).

**Conclusions:**

Optimized design of surgical steps in PPV for MHRD could effectively decrease the rate of iatrogenic retinal tear and shorten the length of operation.

## Background

Macular hole retinal detachment (MHRD) was one of the main reason for uncorrectable visual impairment in high myopic patients [1], which could be a major burden as global prevalence of myopia and high myopia keep increasing [2, 3]. The management of MHRD by pars plana vitrectomy (PPV) was challenging due to the elongation of axial length, presence of posterior staphyloma and severe chorioretinal atrophy. Though myopic eyes had more posterior vitreous detachment (PVD) present in younger patients [4], the rate of residual vitreous cortex adhering to the surface of retina was higher. In another study, the posterior surface of the vitreous cortex was seen to be adhered to the retinal vessels in 50% of high myopic eyes, and in 9.1% in the control group [5]. These characteristics makes it difficult to perform complete PVD induction and clearance of residual vitreous cortex in surgeries for MHRD, which leads to longer operation time. Both macular holes and myopia [6] were risk factors for choroidal detachment in rhegmatogenous retinal detachment, which contributes to the high mobility of detached retina and subsequently high rate of iatrogenic retinal tear.

Among the various techniques for MHRD [7–11], internal limiting membrane (ILM) flap [9] was widely used and achieved satisfactory results. In the present study, we proposed a strategy for its surgical steps optimization and investigated its efficacy in terms of time for surgery and rate of iatrogenic retinal tear.

## Methods

### Participants

We reviewed the medical records of high myopic eyes diagnosed with MHRD receiving PPV from March, 2019 to June, 2020 at Department of Ophthalmology, Xiangya Hospital, Central South University. High myopia was defined as axial length ≥ 26.5 mm or refractive error ≥ 6 diopters. Eyes with concomitant choroidal detachment were also included. Exclusion criteria included retinal detachment associated with peripheral retinal break, idiopathic macular hole without posterior staphyloma, traumatic macular hole, or retinal detachment secondary to vitreoretinal exudations or proliferations. Eyes with other ocular diseases or previous ocular surgeries (esp. PPV or scleral buckle) other than cataract surgery were also excluded. Best corrected visual acuity (BCVA), detailed slit-lamp examination, refractive error, axial length, intraocular pressure, ocular B-ultrasound, color fundus photo and optical coherence tomography (Carl Zeiss Meditec AG, Jena, Germany) scans were performed before and after surgery. This study was approved by the institutional review board of Xiangya Hospital and all procedures adhered to the tenets of the Declaration of Helsinki.

### Surgical methods

Patients were included into either group based on different design of surgical steps. Patients admitted before November 2019 used the conventional design and they were in the routine group. Patients admitted after November 2019 used the optimized design and they were in the experiment group. The surgeries were all performed by the same surgeon (X.W.).

A conventional PPV with ILM flap for MHRD could be divided into following steps: (A) suprachoroidal space fluid was drained through scleral incision if concomitant choroidal detachment was presented; (B) three-port PPV was performed to remove anterior and core vitreous; (C) 4 mg/ml triamcinolone acetonide (TA, Kunming Jida Pharmaceutical Company, Kunming, China) was injected into the vitreous cavity. Unless spontaneously pre-existed, PVD was induced; (D) the area of PVD was extended all the way towards periphery and the vitreous base was excised with scleral indentation; (E) 4 mg/ml TA was injected again to facilitate the clearance of residual vitreous cortex. Epiretinal membrane was removed if presented; (F) the ILM was stained with 1.25 mg/ml indocyanine green (ICG, Dandong Yichuang Pharmaceutical Company, Dandong, China) and peeled to the vascular arcade with preservation of 2 papilla diameter (PD) area around the MH. Gas-fluid exchange was performed to drain subretinal fluid through macular hole. After retinal reattachment, 1–2 ml perfluorocarbon liquid (PFCL, DK-line, Bausch & Lomb, Quebec, Canada) was injected. The infusion was then changed from air to fluid. Residual air bubbles were aspirated by flute needle. The partially peeled ILM was further peeled to the MH periphery in the PFCL from superior, nasal, temporal and inferior. A 360-degree multi-layer ILM flap was made and flipped to cover the MH; (G) retinal laser photocoagulation of possible peripheral degermation areas or iatrogenic breaks was performed. The intraocular fluid and PFCL were aspirated by gas fluid exchange at the optic disc and at the lowest position of the posterior scleral staphyloma with caution to protect the flipped ILM, and the vitreous cavity was filled with silicone oil.

In the routine group, the surgery was finished in the conventional order from A to G. In the experiment group, the surgery was finished in the following order A-B-C-E-F-D-G. In the latter group, the PVD extension and peripheral vitreous cutting was performed after retinal movement was restrained by PFCL.

### Statistical analysis

Statistical analyses were performed using SPSS version 25.0 software (IBM, Armonk, New York). Normally distributed continuous variables were presented as means ± standard deviations. The Snellen visual acuity was converted to the logarithm of the minimal angle of resolution (log- MAR) before analysis. The visual acuity of counting fingers (CF), hand motions (HM), and light perception was converted equal to two, three, and four logMAR units, respectively. Categorical data were compared between the two groups using Fisher’s exact test. Normally distributed continuous data were compared using Student’s t-test. A 2-tailed *P* value of less than 0.05 was considered statistically significant for all analysis.

According to the method proposed by Machin and Campbell [12], the sample size was estimated based on the primary outcome (incidence of iatrogenic retinal break). Assuming that the experiment group is superior to the routine group, the level of significance α = 0.05 (one-sided), power of test 1–β = 0.8, incidence rate in the routine group P_0_ = 37.5%, relative risk RR = 0.18, then the estimated sample size was 19.4. A sample size larger than 19 would be sufficient to detect the difference between the two group.

## Results

A total of 31 eyes (15 eyes in the experiment group and 16 in the routine group) were included in the study. The average age, gender, axial length, refractive error, BCVA before surgery, extent of retinal detachment or rate of concomitant choroidal detachment had no statistically significant difference between the two groups (Table 1).

**Table 1:** Demographic and Preoperative Characteristics

	Experiment Group	Routine Group	*P* value
Number of eyes	15	16	/
Age (years, Mean ± SD)	52.53 ± 8.52	50.93 ± 9.14	0.62
Males	3 (20.0%)	4 (25.0%)	0.75
Females	12 (80.0%)	12 (75.0%)	0.75
Axial Length (mm, Mean ± SD)	29.52 ± 2.33	29.29 ± 2.25	0.78
Refractive Error (diopters, Mean ± SD)	16.30 ± 6.51	15.47 ± 5.40	0.70
Pre-op BCVA (logMAR, Mean ± SD)	1.75 ± 0.65	1.98 ± 0.75	0.36
Retinal detachment within vascular arcade only	4 (26.7%)	4 (25.0%)	0.92
Concomitant choroidal detachment	5 (33.3%)	6 (37.5%)	0.82

SD = standard deviation, Pre-op = before surgery, BCVA = best corrected visual acuity

In both groups, surgery achieved satisfactory results with high rates of retinal attachment (93.3% vs. 87.5%, *P* = 0.60) and MH closure (93.3% vs. 93.8%, *P* = 0.96). In the experiment group, only one case suffered from retinal reattachment failure because of unclosed MH. In the routine group, one case failed due to unclosed MH and the other failed due to proliferative vitreoretinopathy in the periphery. BCVA at 6 months post surgery both improved significantly compared with baseline (in experiment group, 1.16 ± 0.64 vs. 1.75 ± 0.65, *P* < 0.05, in routine group, 1.18 ± 0.43 vs. 1.98 ± 0.75, *P* < 0.05) while no statistically significant difference between the two groups was detected (*P* = 0.91). However, in terms of iatrogenic retinal tear, the experiment group had a much lower rate than the routine group (6.7% vs. 37.5%, *P* = 0.04). The average length of operation was 78.6 ± 18.8 min in the routine group and 64.0 ± 12.1 min in the experiment group (*P* = 0.02) (Table 2).

**Table 2:** Surgical Outcomes

	Experiment Group	Routine Group	*P* value
Retinal reattachment	14 (93.3%)	14 (87.5%)	0.60
Macular hole closure	14 (93.3%)	15 (93.8%)	0.96
Post-op BCVA (logMAR, Mean ± SD)	1.16 ± 0.64	1.18 ± 0.43	0.91
Iatrogenic retinal break	1 (6.7%)	6 (37.5%)	0.04
Length of operation (minutes, Mean ± SD)	64.0 ± 12.1	78.6 ± 18.8	0.02

SD = standard deviation, Post-op = after surgery, BCVA = best corrected visual acuity

## Discussion

The treatment of MHRD in highly myopic eyes has always been a challenge for retinal specialist and various surgery options have been proposed. The present study, for the first time, examined the efficacy of surgical process improvement in reducing iatrogenic retinal tear and shortening operation time. In the experiment group, we witnessed a dramatic drop of iatrogenic retinal tear rate and the time of operation decreased accordingly. The results showed optimization of surgical steps could lead to less complications and possibly better prognosis.

It was reported that induction of posterior vitreous detachment lead to 50% of all iatrogenic retinal tear [13] and risk of developing iatrogenic break seemed to be correlated to adhesion of the posterior vitreous hyaloid [14, 15]. While in pathological myopic patients, the surgical induction of PVD was more difficult with residual vitreous cortex more often presented [16], possibly due to the adhesion caused by presence of proinflammatory and angiogenesis-related cytokines in their vitreous [17]. Ultra-widefield OCT study also found thickened vitreous was observed to adhere to the retinal vessels at multiple points and was accompanied by paravascular abnormalities including lamellar holes in high myopic eyes [5]. Therefore, we suggested to finish operation in the macular area first and extend the area of PVD towards periphery afterwards. The drainage of subretinal fluid from the MH by fluid-gas exchange and sealing the MH with PFCL could achieve retinal reattachment. Under such condition, the extension of PVD towards periphery and vitreous base shaving were performed when retina was attached. The fluttering of retina was much less than PFCL assisted vitrectomy alone. This allows much safer peripheral vitreous cutting, another main cause for iatrogenic break [13].

In the present study, though the rate of iatrogenic retinal tears was higher in the routine group, the rate of retinal reattachment or MH closure was same in the two groups. The reason behind it may be the prompt discovery of iatrogenic break during surgery and proper laser treatment. Iatrogenic retinal tears found after surgery usually causes rhegmatogenous retinal detachment and requires further surgery [14, 18, 19].

The average length of operation in the experiment group was shorter because of the following reasons: (1) the extension of PVD and cutting of peripheral vitreous was performed after retinal reattachment was achieved. It allows relatively high vacuum aspirating pressure settings and subsequently more efficient vitrectomy, esp. when choroidal detachment or ciliary body detachment was combined; (2) if no iatrogenic retinal break was presented, no further injection of PFCL to the periphery was needed. The risk of PFCL bubbles caused by fluid flow [20] might be reduced and the time for complete PFCL removal would be shortened; 3)lower rate of iatrogenic retinal tear reduced the time for laser.

Longer duration of surgery was associated with higher risk of intentional corneal epithelial debridement in surgery and corneal epithelial defects [21, 22] after surgery. Shortening the operation time by surgical process improvement might increase the overall satisfaction of patients.

The limitation of the study included: (1) small samples. The sample size was small and larger samples may help better show the differences between the experiment group and routine group, esp. in terms of complications. (2) retrospective nature. The study was retrospective and the choice of which surgical process to use was related to the time of patient admission. Though the baseline characteristics between the two groups were similar, a randomized controlled trial would be more persuasive.

## Conclusions

In summary, optimized design of surgical steps in PPV with ILM flap for MHRD in pathological myopia could decrease rate of iatrogenic retinal break and shorten the length of operation. Exploring new surgical methods is essential, while process improvement of a conventional surgery could also be important. Standardized surgical steps may lead to less complications and better prognosis.

## Data Availability

All data generated or analysed during this study are included in this published article.

## List of abbreviations

PPV: pars plana vitrectomy
ILM: internal limiting membrane
MHRD: macular hole retinal detachment
BCVA: best corrected visual acuity
PVD: posterior vitreous detachment
TA: triamcinolone acetonide
ICG: indocyanine green
PFCL: perfluorocarbon liquid
log-MAR: logarithm of the minimal angle of resolution
CF: counting fingers (CF)
HM: hand motions
